# Publishing case studies in health sciences librarianship

**DOI:** 10.5195/jmla.2017.212

**Published:** 2017-04

**Authors:** Katherine G. Akers, Kathleen Amos

While most issues of the *Journal of the Medical Library Association (JMLA)* contain one or two case studies, the *JMLA* editorial team is pleased to note that the current issue contains six case studies, highlighting a wide range of library-driven initiatives to support health sciences research and education. Briefly, Surkis et al. [1], Read et al. [2], and Brandenburg and Garcia-Milian [3] detail efforts to strengthen the research data management and analysis capabilities of biomedical researchers. von Isenburg et al. [4] describe a writing boot camp aimed at helping health professionals prepare their work for scholarly publication. Also, Marshall and Hobbs [5] narrate the creation of an online digital collection of hospital historical materials, and Mages and Lohr [6] discuss the use of a controversial archival text to enhance the education of medical students.

This unusually large number of case studies published in a single issue of the *JMLA* provides us an opportunity to remind readers of the value of case studies to health sciences librarianship and to offer advice to authors on how to write a strong case study.

## THE VALUE OF CASE STUDIES TO HEALTH SCIENCES LIBRARIANSHIP

Borrowing the framework of evidence-based medicine (EBM), evidence-based librarianship (EBL) can be considered “librarianship in which management and service decisions are constructed from research-based, strong evidence” [7]. As in EBM, the levels of evidence for EBL can be thought of as a hierarchy, with knowledge syntheses and experimental studies near the top and descriptive studies and expert opinion near the bottom [8, 9].

As a type of descriptive study, case studies recounting particular library experiences or initiatives occupy a low position in the EBL hierarchy of evidence, because they are anecdotal, not generalizable, and prone to various types of bias, including positive outcome bias [8]. Therefore, case studies provide only weak evidence, at best, for making decisions [7].

However, we believe that case studies still have value for health sciences librarianship for several reasons. First, case studies can communicate timely and innovative approaches to librarianship and can generate hypotheses that can be tested by future studies employing more rigorous research designs. This ongoing cycle serves to build the evidence base essential for the continuous improvement of our practice.

Second, case studies are a conduit for detailed information sharing. Many of us engage in similar activities at our workplaces, yet we often reinvent the wheel even though we might benefit from knowing more about how colleagues at other institutions have accomplished the same tasks. By thoroughly describing the planning and implementation of specific library projects, case studies can impart wisdom and insight to readers, giving them a leg up in their work on similar projects.

**Figure 1:**
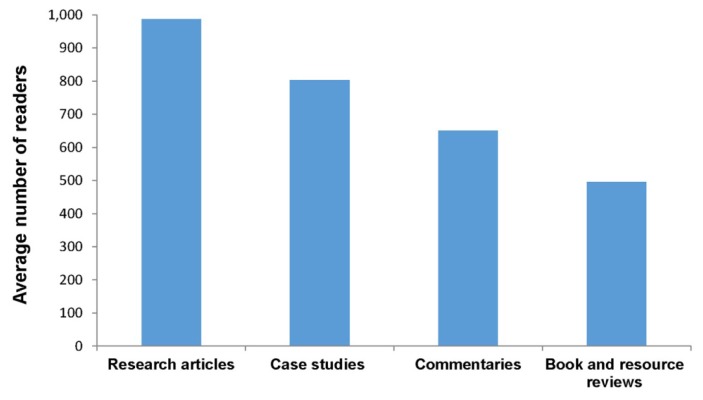
Average number of readers (measured by the number of unique user Internet protocol addresses) of different types of articles published in the *Journal of the Medical Library Association (JMLA)* The usage of articles published in 2015 was assessed at the end of 2016.

Third, case studies, which are essentially stories, are often quite interesting to read, sometimes more so than research articles. In fact, case studies are among some of the most highly read articles in the *JMLA*. Specifically, we found that case studies published in the *JMLA* in 2015 had been accessed by approximately 800 readers, on average, by the end of 2016 (Figure 1). Therefore, case studies appear to be well liked by readers and can potentially have a large impact on the practice of health sciences librarianship.

**Figure 2:**
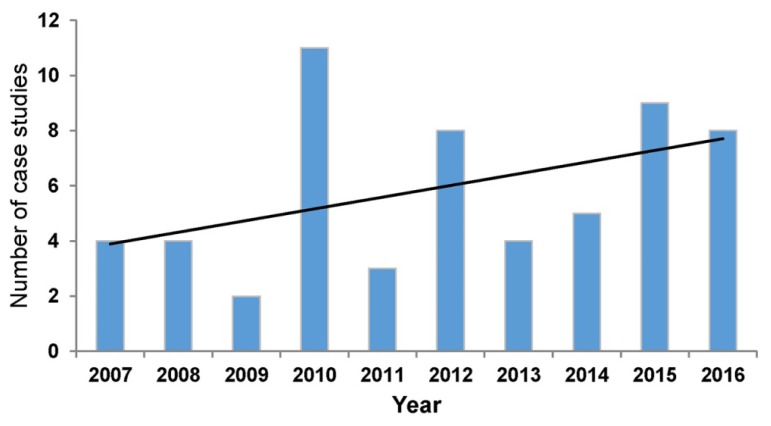
Total number of case studies published in the *JMLA each year* Black line indicates an increasing linear trend in the number of case studies published over time.

Fourth, although many of us face expectations to engage in scholarship, we often do not have the time, resources, or training needed to perform proper research studies. Publishing a case study is an alternative way for practicing librarians to engage in scholarly discourse, contribute to the literature supporting the profession, and add to their curriculum vitae (CV). Despite considerable year-to-year variation, we have seen an increasing trend in the number of case studies published annually in the *JMLA* over the last decade (Figure 2), which underlines our commitment to advancing both the theory and practice of health sciences librarianship.

## WHAT MAKES A GOOD CASE STUDY?

Case studies published in the *JMLA* describe an improvement in services or an innovative resolution to a problem or issue that advances health sciences librarianship. They are brief (1,000–2,500 words), peer-reviewed articles that show evidence of the success or failure of the described project. To prospective authors who are interested in publishing a case study in the *JMLA,* we offer the following advice.

### Provide broad context

Providing broad context to your case study will help capture interest and convince readers of its importance to health sciences librarianship in general and their own practice in particular. Strong case studies not only explain an institution-specific need, but also situate that need within larger problems or current trends at national or global levels.

### Specify your purpose

Strong case studies provide sufficient background information about the institution or local environment to clearly convey why a change in library workflow or services was needed or opportune at that moment in time. Similar to describing the aims of a research study, authors of case studies should state the specific purpose of the described initiative and its intended outcome.

### Provide detailed information

Case studies that provide detailed description of the planning and implementation of an initiative are more interesting and useful than case studies that provide only a general overview of a project. Readers want to know not only what you did, but also how you did it. Try to adopt a reader’s perspective: What information would be most useful to others who might wish to replicate your results?

### Report objective data

Strong case studies report evaluative data that allow readers to independently judge the success or failure of the initiative. Although subjective opinions can be informative, objective data such as usage statistics or learning outcomes provide stronger evidence of how the initiative benefitted library patrons. Describe both positive and negative feedback or outcomes when applicable.

### Discuss lessons learned

Strong case studies not only describe the planning and implementation of an initiative, but also discuss lessons learned in the process. Consider questions such as: What unforeseeable problems arose? What would you have done differently? What factors should be considered to increase the likelihood of a successful outcome? Readers have much to gain from your hindsight.

### Relate your case study to previous literature

Strong case studies contribute to the scholarly discourse. While your case study will not include a formal literature review, it is important to read relevant literature and to cite and discuss pertinent articles in your case study. Explain how your case study adds to the literature and advances the knowledgebase and/or practice of health sciences librarianship.

On behalf of the *JMLA*, we look forward to receiving your case study submissions and continuing to disseminate innovative approaches to meeting the health-related information needs of clinicians, researchers, students, and community members.
